# The Immunogenicity of the C Fragment of Tetanus Neurotoxin in Production of Tetanus Antitoxin

**DOI:** 10.1155/2018/6057348

**Published:** 2018-12-31

**Authors:** Rui Yu, Chong Ji, Junjie Xu, Denghai Wang, Ting Fang, Yue Jing, Clifton Kwang-Fu Shen, Wei Chen

**Affiliations:** ^1^Beijing Institute of Biotechnology, 20 Dongdajie Street, Fengtai District, Beijing, China; ^2^Gaotai Tianhong Biochemical Technology Development Co. Ltd., Gaotai, Gansu, China; ^3^Jiangxi Institute of Biological Products, Jian, Jiangxi, China; ^4^Shenzhen Qianhai Tianzheng (SQT) Biotechnology Ltd., Shenzhen, Guangdong, China; ^5^Shenzhen JinRuiFeng (Golden Harvest) Biotechnology Ltd., Shenzhen, Guangdong, China

## Abstract

The demand of tetanus antitoxin (TAT) as tetanus treatment in developing and underdeveloped countries is still great since it is relatively easy to achieve and affordable. However, there are still issues in the preparation of highly effective TAT with tetanus toxoid (TT) as the immunogen. The tetanus toxin native C-fragment (TeNT-Hc) retains many properties and it is a very promising candidate for the development of tetanus human vaccine. In this study, we tested the immunogenicity of TeNT-Hc in the preparation of tetanus antibodies, by TeNT-Hc alone or in different combinations with TT. The antibody titers and components in horse serum or plasma in different groups were analyzed and compared with those immunized by the conventional TT and it showed comparability with the results of traditional methods. The plasma efficacy and* in vivo* tetanus toxin neutralization were also tested. After two stages of immunizations, the average potency in plasma of all groups reached more than 1,000 IU / mL except that in group 4. In group 5, the first two basic immunizations with TT and the subsequent immunizations with TeNT-Hc, it showed slightly higher antibody titers and potency. This study demonstrated that TeNT-Hc is a safe, effective, and yet easy-to-produce low-cost immunogen and suitable for TT replacement in tetanus antitoxin production.

## 1. Introduction

Tetanus is an acute, lethal infectious disease and neurological disorder with fatality rate up to 40% [1]. Any injury or trauma has the possibility of getting* Clostridium tetani* (*C. tetani*) infection that causes tetanus toxin production [2]. Tetanus antibodies are the only effective intervention for preventing and treating tetanus on the first two-week period of infections [3, 4]. Tetanus antitoxin (TAT) and tetanus immunoglobulin (TIG) are currently available on the market [5, 6]. TIG, purified human tetanus antibodies, is manufactured from plasma of blood donors immunized with hepatitis B vaccine and then tetanus toxoid (TT) [7]. Due to its human origin, TIG can be applied directly without skin test. However, since it is a human blood product, potential risk of infecting human viruses, such as hepatitis C, AIDS, and other infectious diseases, still remains. In addition, due to source restrictions and unstable supplies, TIG products are generally rather expensive have hard-to-find and have only 20% or lower efficacy per vial compared to TAT, which requires multiple doses. Therefore, it is very difficult for developing and underdeveloped countries to afford TIG for the prevention and treatment of tetanus [8]. On the contrary, utilization of TAT as tetanus treatment in those countries is relatively easy to achieve and affordable. Thus, we can predict it will continue being in clinical applications in the near future [9].

TAT is made of toxin-neutralizing immunoglobulin fragments F (ab')_2_, extracted, and purified from tetanus toxoid-immunized horse blood [10]. At present, there are still issues in the preparation of highly effective TAT: (1) due to its high toxicity, it is difficult to purify raw tetanus toxin and afford its high-purity form, even after formaldehyde inactivation/detoxification. During the initial immunization and subsequent hyperimmunization processes, interference of impurities within TT antigen results low-grade antibodies against tetanus toxin; (2) since TT still has residue toxicity, frequent and high dose injection of TT antigen into blood-harvesting horses induces the gradual hepatic degeneration and necrosis of their liver cells. This effect is generally quite visible after prolonged, repeated immunization cycle. During this time, the horse liver continues enlarging and deteriorating, and finally it ruptures and causes internal bleeding and the death of the horse; (3) the amount of TT used in horse immunization processes is considerably large and results in rather high cost. Therefore, antigen quality improvement, toxicity reduction, and lower production costs are all essential to achieve better TAT [11].

Tetanus toxin is an enormously potent neurotoxin secreted by the anaerobic bacterium* C. tetani*. After collecting culture supernatants and inactivating with formaldehyde, tetanus toxoid (TT) is simply harvested by filtration. Despite its good immunogenicity in horse, inactivated toxoid generally contaminates with residual formaldehyde which is still toxic. Additionally,* C. tetani* can form spores that resist heat and chemical treatment and post certain risks in TT production. Furthermore, inactivation step with formaldehyde sometimes could not guarantee complete detoxification of tetanus toxin that could be harmful to the horses [12].

The tetanus toxin native C-fragment (TeNT-Hc) retains many properties such as intact binding to gangliosides, immunogenic potency comparable to native toxin, low toxicity, and low allergenicity [13]. It is a very promising candidate for the development of tetanus subunit vaccines and genetic engineering vaccines, which have been used to in Phase I clinical trial as replacement of TT, vaccine conjugates with bacterial and viral vectors, mucosal vaccines and many more [14–17]. Our group has successfully developed a recombinant human tetanus vaccine with TeNT-Hc as an antigen in the past few years [18–20]. This study aimed at animal immunogenicity and toxicological pharmacology demonstrated that TeNT-Hc is a safe, effective, and yet easy-to-produce low-cost immunogen and suitable for TT replacement in tetanus antitoxin production.

In this study, we tested the immunogenicity of TeNT-Hc in the preparation of tetanus antitoxins, by TeNT-Hc alone or in different combinations with TT, to exploit its potential as a replacement immunogen of TT.

## 2. Materials and Methods

Animals: Adult male horses (4 to 10 years old, 250-400 kg body mass), without tetanus natural antibodies, were purchased from Datong area in Qinghai Province, China. ICR mice (17-19g, male and female) were purchased from Hunan SJA Laboratory Animal Co., Ltd. The horse and mice studies were carried out in accordance with the recommendations of SQT Biotech Antitoxin Production Council on Equine Welfare Guidelines (GS-P009-01 and GS-P001-01). The protocol was approved by the SQT Biotech Antitoxin Production Council (OS-P003-01 to OS-P008-01, GS-P006-01 and GS-P015-01). All animals used in this study were raised under humanitarian conditions with free access to food and water. All efforts were made to minimize suffering. After injection, mice were followed for the five days to check for any signs of paralysis or death. Loss of righting reflex was used as the humane end point of the experiment. Mice were monitored three times a day for their condition and for the occurrence of end point.

### 2.1. Materials

**Figure 1:**
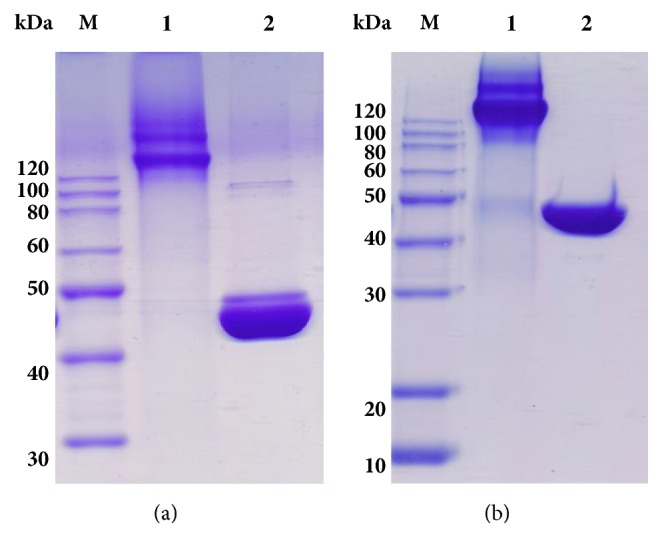
Nonreducing (a) and reducing (b) SDS–PAGE analysis of TT and TeNT-Hc. Lane M: molecular weight markers; lane 1: TT; lane 2: TeNT-Hc.

*Incomplete Freund's Adjuvant*. Liquid paraffin (Shanghai Zhongqin Chemical Reagent Co., Ltd.) and lanolin (Medical grade, China Huating Lanolin Plant) were mixed (volumetric ratio of 2: 1) to afford the adjuvant.* Antigen and Adjuvant*. TeNT-Hc (molecular weight 45 kD, prepared by the Department of vaccine and antibody engineering, Beijing Institute of Biotechnology) was mixed with incomplete Freund's adjuvant into a water-in-oil emulsion with protein content of 0.625 mg/mL. Tetanus toxoid (TT) purchased from Chengdu Olymvax Biopharmaceuticals, China, was mixed with incomplete Freund's adjuvant into a water-in-oil emulsion with a protein content of 0.625 mg/mL. A mixed antigen solution was prepared by mixing TeNT-Hc and TT with incomplete Freund's adjuvant with incomplete Freund's adjuvant into a water-in-oil emulsion with a protein content of 0.625 mg/mL. The SDS-PAGE electrophoresis analysis of TeNT-Hc and TT is shown in Figure 1. In comparison with TT, TeNT-Hc was shown as single component and has higher purity and smaller molecular weight.* Secondary Antibody*. HRP-conjugated anti-horse IgG and IgM as secondary antibodies was purchased from Abcam.* Standard*. Standard tetanus toxins and anti-tetanus serum were purchased from the China National Institutes of Food and Drug Control.* Borate Buffer*. The borate solution was made of 1L of water, 8.5g of NaCl, 4.5g of H_3_BO_3_, and 0.5g of Na_2_B_4_O_7_·10H_2_O and adjusted pH to 7.0-7.2.

### 2.2. Horse Grouping, Immunization Schedule, and Serum Plasma Separation

**Figure 2:**
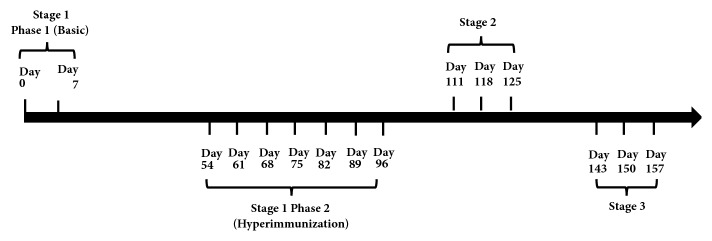
The summary of the immunization schedule and the shots.

Eighteen horses, with no natural antibodies against TeNT, were divided into 6 groups (3 horses each group) according to age, body weight, and health condition. Following the immunization schedule, different doses of TeNT-Hc, TT or mixed antigens (Mix Ag) were injected intramuscularly into the neck and back of horses. A three-stage immunization schedule was applied: (1) the first stage contains two basic immunization phases. The first phase is basic immunization with two shots and 7-day interval. Before the second phase, there is a rest of 56 days. The second phase is hyperimmunization with 7 shots and 7-day interval; (2) the second stage begins after a rest of 16 days with 3 shots and 7-day interval; (3) the third stage begins after a rest of 18 days with 3 shots and 7-day interval. The specific types of antigens and immunization dose administrated was summarized in Table 1. The schedule and the shots were summarized in Figure 2. In different stages/phases of immunization schedule, various samples of corresponding horse blood were collected and analyzed. Horse blood samples were collected from the jugular veins and the corresponding plasma was separated using a modified human blood apheresis machine. The corresponding serum was collected after the horse blood clotted and centrifugation.

### 2.3. SDS-PAGE Analysis of Plasma Antibodies

The plasma collected after the final immunization of the third stage from each group or collected after routine TT immunization was diluted 10 times with normal saline. After being added into 2 × loading buffer, the samples were detected by a 12% SDS-PAGE and the differences plasma components between different immune groups were analyzed.

### 2.4. Antibody Level Analysis

Horse serum and plasma were collected respectively at the end of the first stage (basic immunization, hyperimmunization), the second stage, and the third stage. The anti-TT and anti-TeNT-Hc antibody titers were determined by ELISA. The specific method was summarized as following: Each well on the 96-well ELISA plate (Costar) was coated with 2 *μ*g / mL TT or TeNT-Hc at 4°C overnight, and washed 4 times with PBST (PBS + 0.1% Tween-20). Horse serum or plasma was diluted (1: 20000 v/v for IgG detection or 1: 500 for IgM detection), incubated at 37°C for 1 h, washed 4 times in PBST for 5 min, added 1: 100,000 dilution of HRP-anti horse IgG (1:100,000 dilution) or HRP-anti horse IgM (1: 20000 dilution), incubated at 37°C for 40 min, washed with PBST 4 times, and finally added chromogenic solution (TMB, Sigma). After color development, 2 M H_2_SO_4_ was applied to stop the reaction and the final readout was performed at 450 nm.

### 2.5. Agar Diffusion Test to Determine Plasma Antibody Titer

2.25g of agarose was added 150 mL of purified water. It was heated to boil and poured into petri dishes. After cooling, 9 sample reservoirs were made by a hole puncher (one hole in the center with equal distance to the rest eight holes). To the center hole 100 *μ*L of TeNT-Hc (0.17mg/mL) was added and, for the rest of holes, in the clockwise fashion, 100 *μ*L of serial diluted immunized horse sera (v/v: 1/ 5, 1/10, 1/20, 1/40, 1/80, 1/160, 1/320, and 1/640) was added, respectively. The loaded petri dish was placed in a moisture control chamber and incubated at 37°C for 48 hours before recording the resulting precipitation lines.

### 2.6. Flocculation Method to Determine Plasma Efficacy

According to the method in Chinese Pharmacopoeia (2015 edition, Method 3506), different volumes (100 Lf/mL) of tetanus toxin standard solution were precisely-measured and added to the corresponding reaction tubes. Then diluted horse plasma (1 mL) was added quickly into those tubes and mixed thoroughly. The tubes were immersed in a water bath (45-50°C) and observed closely, and the volume of standard solution for first occurrence of flocculation was recorded. After repeating three times, the horse plasma efficacy (in flocculation unit (Lf/mL) =* V* ×* n* × 100 [*V* is the volume of the tetanus toxin standard solution used in the first flocculation (mL);* n* is the dilution of the horse plasma]) can be determined.

### 2.7. *In Vivo* Tetanus Toxin Neutralization Test

According to the method in Chinese Pharmacopoeia (2015 edition, Method 3508), 0.2mL of the tetanus antitoxin standard (*ca* 0.5 IU/mL) and different concentrations of horse plasma (diluted by borate buffer) were mixed with 0.2mL of tetanus toxin standard, respectively. The mixtures were incubated at 37°C for 1 h and were then injected into mice intraperitoneally. There were three mice in each group and each group was observed at least twice a day for the first five days. The control group should all die within 72 to 120 hours. The efficacy of the horse serum was evaluated as the highest dilution which is most likely to die of the same symptoms as the control mice.

### 2.8. Statistical Analysis

Unpaired two-tailed Student's* t*-test was used to determine the significance of the differences in antibody titers and neutralizing potency between the groups. Probability (*P*) values <0.05 were considered to be significant and marked as *∗*.* P*<0.01 was considered to be very significant and marked as *∗∗*.

## 3. Results

### 3.1. Comparison of Plasma Composition after Different Immunization Methods

**Figure 3:**
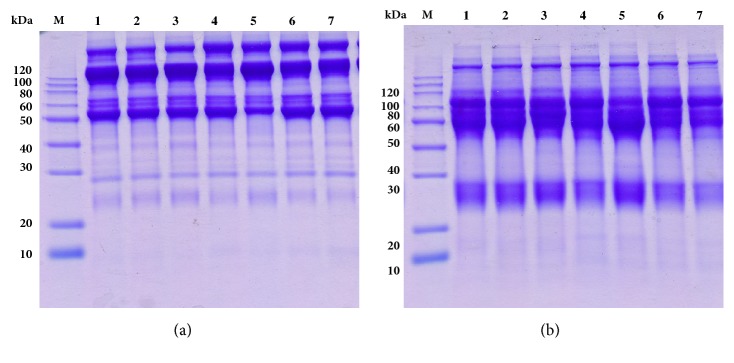
Nonreducing (a) and reducing (b) SDS–PAGE analysis of horse plasmas. Lane M: molecular weight markers; lanes 1 to 6: plasma from horses in groups 1 to 6; lane 7: plasma from routine TT immunized horses.

As shown in Figure 1, TeNT-Hc was shown as single component and has higher purity and smaller molecular weight in comparison with TT. However, SDS-PAGE (Figure 3) showed no significant difference between the main components (as well as antibodies) in the horse plasma prepared by different immunization methods (lanes 1-6) and those immunized by TT (lane 7). The molecular weight and proportion of the main components of each group are very similar.

### 3.2. Anti-Tetanus Toxin Antibody Levels Induced by TeNT-Hc

**Figure 4:**
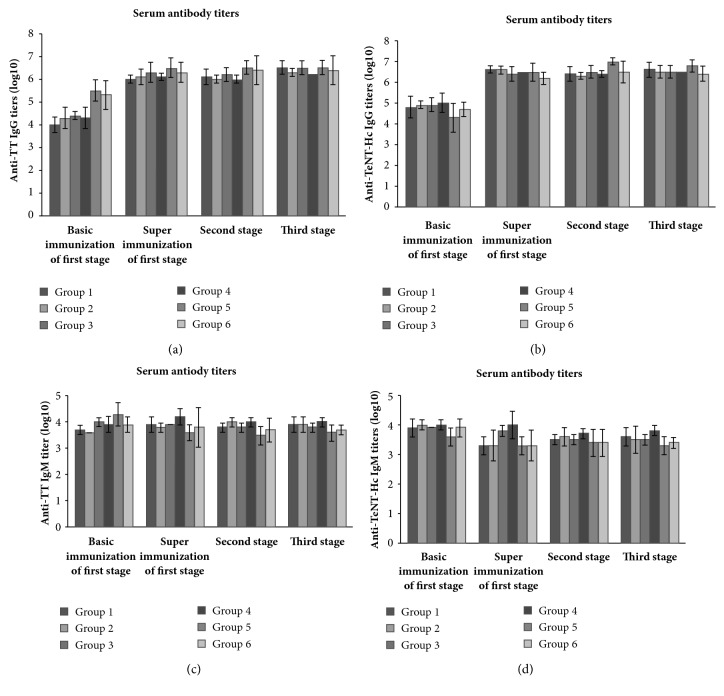
**Serum antibody titers after different immunization stages**. (a) Anti-TT IgG titers in serum; (b) anti-TeNT-Hc IgG titers in serum; (c) anti-TT IgM titers in serum; (d) anti-TeNT-Hc IgM titers in serum. Unpaired two-tailed Student's* t*-test was used to determine the significance of the differences in antibody titers between the groups.* P* values ≤ 0.05 were considered to be significant.

Serum titers of anti-TT, anti-TeNT-Hc IgG or IgM induced by TeNT-Hc alone or TeNT-Hc and TT co-immunization were tested. As shown in Figure 4, the titers of anti-TT IgG in serum of groups 5 and 6 were significantly higher than other groups one week post the basal immunization. However, further along the immunization schedule, there was no significant difference (*p*≥0.05) of anti-TT IgG antibody titers in serum among the groups except group 4 one week after the first, second, and third immunization. The lowest anti-TT IgG antibody titer in group 4 has significant difference with the highest antibody titer in group 5 (*p *<0.05). Likewise, there were no significant difference in anti-TeNT-Hc IgG antibody titer, anti-TT, and TeNT-Hc IgM antibody titers between groups observed in immunization progress (*p*> 0.05). All immune methods can induce humoral immunity based on IgG antibody.

**Figure 5:**
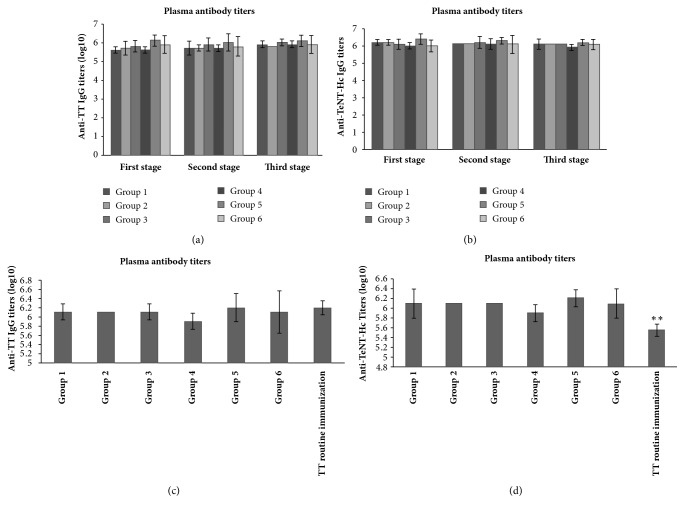
**Plasma antibody titers after different immunization stages**. (a) Anti-TT IgG titers in plasma from group 1-6; (b) anti-TeNT-Hc IgG titers in plasma; (c) anti-TT IgG titers in plasma after three stages immunization and TT routine immunized plasma; (d) anti-TeNT-Hc IgG titers in plasma after three stages immunization and TT routine immunized plasma. Unpaired two-tailed Student's* t*-test was used to determine the significance of the differences in antibody titers between the groups.* P* values ≤ 0.05 were considered to be significant.* P*<0.01 was considered to be very significant and marked as *∗∗*.

In addition to serum antibodies, antibody titers of anti-TT and anti-TeNT-Hc IgG in horse plasma one week after each stage of immunization were also studied (Figure 5). As the whole, the anti-TT IgG antibody titer in group 5 was generally higher than those in other groups and significantly higher than those in groups 1 and 4 after the first stage immunization (*p *<0.05). However, there was no significant difference of anti-TT IgG titers among the groups after the second and the third stage immunization (*p*≥0.05). The titer of anti-TeNT-Hc IgG antibody in group 4 was slightly lower and significantly lower than those in group 5 after the first and third stage immunization (*p*<0.05). No significant difference of anti-TeNT-Hc IgG titers was found among the other groups (*p*≥0.05). The anti-TT and anti-TeNT-Hc IgG titers in plasma after three stages of immunization with TeNT-Hc were compared with those collected after routine immunization with TT. The levels of anti-TT IgG antibodies among different groups had no significant difference (*p*≥0.05). However, the titers of anti-TeNT-Hc IgG antibody in the plasma of the TT conventional immunized group were significantly lower than those of the six groups of horse plasma immunized with TeNT-Hc (*p *<0.01).

### 3.3. Specific Antibody Levels in Horse Plasma Tested by Agarose Diffusion

**Figure 6:**
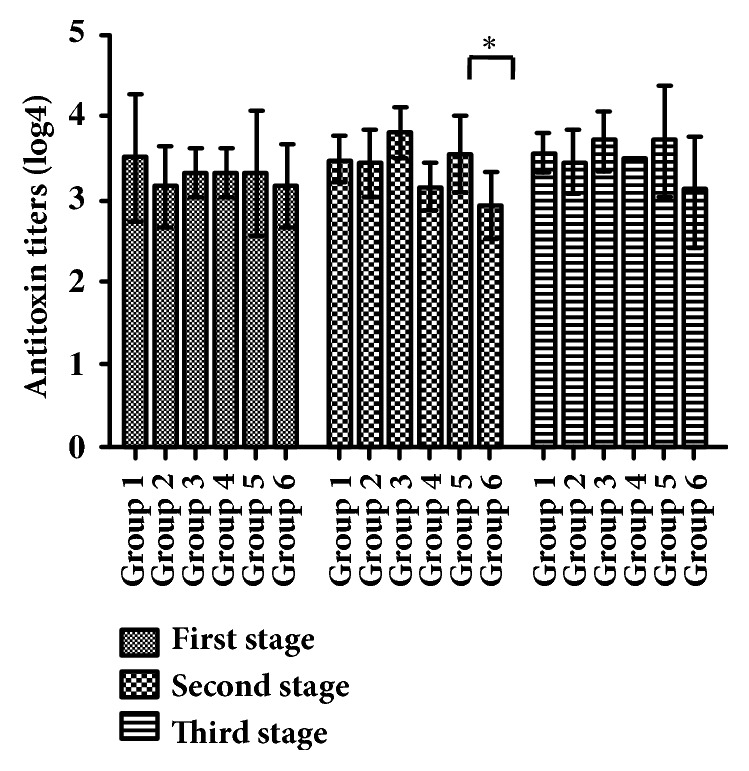
**Plasma antibody titers tested by agarose diffusion method**. Unpaired two-tailed Student's* t*-test was used to determine the significance of the differences in antibody titers between the groups.* P* ≤ 0.05 was considered to be significant and marked as *∗*.

The titers of antitoxins specific binding to TeNT-Hc in different groups of horse plasma were evaluated of at different immunization stages by an agarose diffusion method. According to the result shown in Figure 6, the titers between group 3 and 4 had significant difference after the second immunization (*p*<0.05). There was no significant difference in antibody titers among groups after the first and the third stage of immunization (*p*≥0.05).

### 3.4. Antitoxin Titers in Plasma Measured by Flocculation

**Figure 7:**
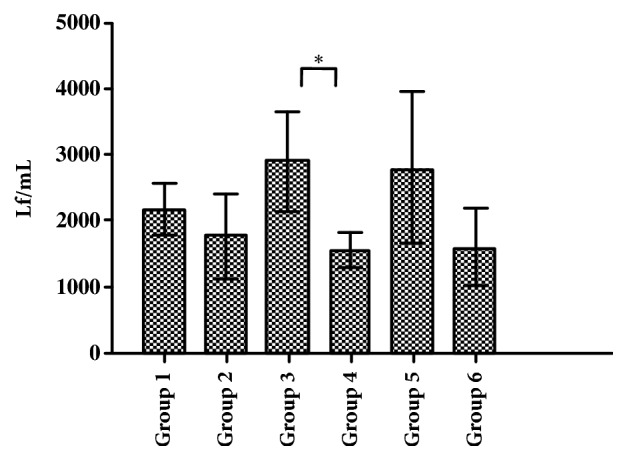
**Antitoxin titers in plasma measured by flocculation**. Unpaired two-tailed Student's* t*-test was used to determine the significance of the differences in antibody titers between the groups.* P*≤ 0.05 was considered to be significant and marked as *∗*.

In our preliminary experiments, the potency of TeNT-Hc with a protein concentration of 0.17 mg / mL was determined to be equivalent to the titer of tetanus toxoid (TT) of 100 Lf / mL. The potency of each group after three immunization stages was compared by using the TeNT-Hc (0.17 mg / mL) as the antigen. As shown in Figure 7, the antitoxin flocculent units in group 3 and group 5 were slightly higher than those in other groups. The flocculent units in group 4 were significantly lower than those in group 3 (*p* <0.05). There was no significant difference among groups 1, 2, 3, 5, and 6 (*p*≥0.05).

### 3.5. *In Vivo* Neutralization of the Antitoxin in Mice

**Figure 8:**
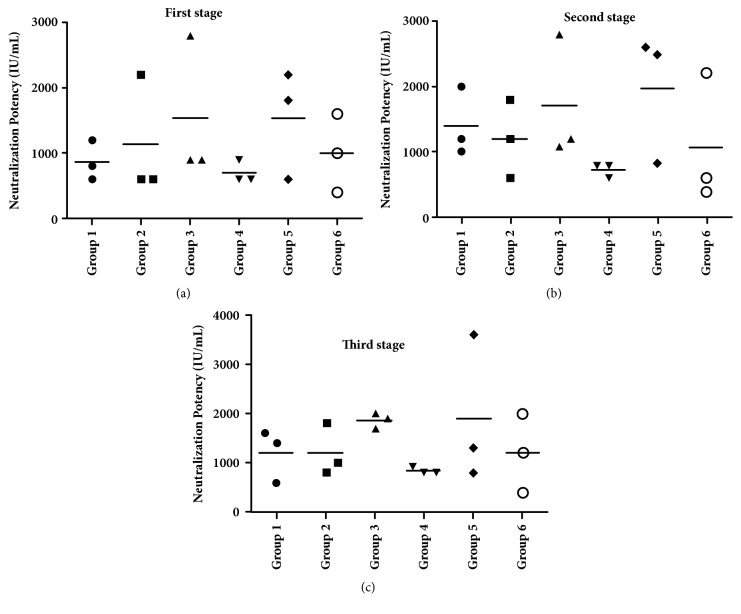
**Neutralizing potency of the corresponding antibodies in plasma from group 1-6**. (a) Plasma collected after the first immunization stage; (b) plasma collected after the second stage; (c) plasma collected after the third stage. Unpaired two-tailed Student's* t*-test was used to determine the significance of the differences in antibody titers between the groups.

The* in vivo* neutralization test in mice was used to test the antitoxin potency of the plasma in each group one week after each stage of immunization. As shown in Figure 8, at one week after the first stage of immunization, the average titers of the plasma antitoxins in groups 2, 3, 5, and 6 were all higher than 1000 IU /mL, while the average titers of in groups 1 and 4 were less than 1000 IU /mL. One week after the second and third stage of immunization, the average antitoxin in each group was higher than 1000 IU/mL except that in group 4. According to statistical analysis, there was no significant difference among the antitoxin titers in groups 1, 2, 3, 5, and 6 (*p*≥0.05).

## 4. Discussion and Conclusion

As an effective immunogen, TT has played a critical role in the preparation of TAT. However, there were still inherent drawbacks of using TT: the production scale of toxin and bacterial strains were limited due to safety concern, the formaldehyde detoxification is generally incomplete, and the large-scale waste could cause environmental issues; the repeated immunization with considerable high-dose to the blood-harvesting horses will, sooner or later, induce the liver toxicity, resulting in reduced product quality and increased production costs. TeNT-Hc, the nontoxic fragment from TT and recombinant expressed in* E. coli*, has good immunogenicity, is low-cost, is easy to scale up, and is purified, not mention it is completely safe and nontoxic to both humans and horses. TeNT-Hc as a candidate for human tetanus vaccine is currently well underway and sued as a potential replacement of TT in TAT production. In this paper, using an established immunization procedures and doses, the* in vivo* immunogenicity of TeNT-Hc in horses was tested alone or in combination with TT antigen. The antibody titers in serum or plasma in different groups were compared with those immunized by the conventional TT and it showed very similar results from those using TT immunization. After additional two stages of immunization, the average potency in plasma of all groups reached more than 1,000 IU / mL except that in group 4. With refining the immunization methods and adjust the dosage, we have confidence that the results could easily exceed the potency required for tetanus antitoxin production. The purpose of this study is to evaluate the possibility of TeNT-Hc as an immunogen to replace TT in the tetanus antitoxin production. The difference in immune programs will affect the titers of antibodies and antitoxins. The immunization program used in this study is a routine and optimized procedure for the preparation of tetanus antitoxin by TT immunization. For TeNT-Hc, this program may be not the optimal program and it is necessary to further explore the best immunization program in the follow-up study.

As the experimental results show, there was no significant difference between the groups. In group 5, the first two basic immunizations with TT and the subsequent immunization with TeNT-Hc, it showed slightly higher antibody titers and potency. In group 4, which utilized the sequence of hyperimmunization with TT and the rest immunization with TeNT-Hc, it resulted in lower antibody titers and potency. The result that there was no significant difference in immunogenicity between the different dose groups and the different antigen combination groups may indicate that the lowest dose of TeNT-Hc used in the experiment was sufficient for the preparation of antitoxin. However, due to the small number of samples and large variations between the individual horses, a larger scale experiment is warranted to figure out the optimal immunization program. By optimizing the immunization schedule and the corresponding doses, TeNT-Hc may even show better immunogenicity.

Without the accumulated liver toxicity from TT immunization, TeNT-Hc positions itself as a completely nontoxic recombinant replacement while retaining all the effectiveness of TT, as a long-term safety study could easily reveal. In conclusion, this study further confirmed the validity of using TeNT-Hc to be replaced or used in conjugation with TT to produce TAT.

## Figures and Tables

**Table 1:** Horse groups and immunization schedule and doses.

**Schedule**	**Number of shots**	**Group 1 (mg)**	**Group 2 (mg)**	**Group 3 (mg)**	**Group 4 (mg)**	**Group 5 (mg)**	**Group 6 (mg)**	**Routine TT immunization (mg)**
**Stage 1 Phase 1 (Basic)**	1	TeNT-Hc 0.625	TeNT-Hc 1.25	TeNT-Hc 0.625	TeNT-Hc 0.625	TT 0.625	Mix Ag 0.625	TT 0.625
	2	TeNT-Hc 1.875	TeNT-Hc 3.75	TeNT-Hc 1.875	TeNT-Hc 1.875	TT 1.875	Mix Ag 1.875	TT 1.875

**Stage 1 Phase 2** **(Hyperimmunization)**	1	TeNT-Hc 1.25	TeNT-Hc 1.875	TT 1.25	TeNT-Hc 1.25	TeNT-Hc 1.25	Mix Ag 1.25	TT 1.25
	2	TeNT-Hc 2.5	TeNT-Hc 3.75	TT 2.5	TeNT-Hc 2.5	TeNT-Hc 2.5	Mix Ag 2.5	TT 2.5
	3	TeNT-Hc 3.75	TeNT-Hc 5.625	TT 3.75	TeNT-Hc 3.75	TeNT-Hc 3.75	Mix Ag 3.75	TT 3.75
	4	TeNT-Hc 5	TeNT-Hc 7.5	TeNT-Hc 5	TT 5	TeNT-Hc 5	Mix Ag 5	TT 5
	5	TeNT-Hc 5	TeNT-Hc 7.5	TeNT-Hc 5	TT 5	TeNT-Hc 5	Mix Ag 5	TT 5
	6	TeNT-Hc 7.5	TeNT-Hc 11.25	TeNT-Hc 7.5	TeNT-Hc 7.5	TeNT-Hc 7.5	Mix Ag 7.5	TT 7.5
	7	TeNT-Hc 10	TeNT-Hc 15	TeNT-Hc 10	TeNT-Hc 10	TeNT-Hc 10	Mix Ag 10	TT 10

**Stage 2**	1	TeNT-Hc 3.75	TeNT-Hc 3.75	TeNT-Hc 3.75	TeNT-Hc 3.75	TeNT-Hc 3.75	Mix Ag 3.75	TT 3.75
	2	TeNT-Hc 7.5	TeNT-Hc 7.5	TeNT-Hc 7.5	TeNT-Hc 7.5	TeNT-Hc 7.5	Mix Ag 7.5	TT 7.5
	3	TeNT-Hc 15	TeNT-Hc 15	TeNT-Hc 15	TeNT-Hc 15	TeNT-Hc 15	Mix Ag 15	TT 15

**Stage 3**	1	TeNT-Hc 3.75	TeNT-Hc 3.75	TeNT-Hc 3.75	TeNT-Hc 3.75	TeNT-Hc 3.75	Mix Ag 3.75	TT 3.75
	2	TeNT-Hc 7.5	TeNT-Hc 7.5	TeNT-Hc 7.5	TeNT-Hc 7.5	TeNT-Hc 7.5	Mix Ag 7.5	TT 7.5
	3	TeNT-Hc 15	TeNT-Hc 15	TeNT-Hc 15	TeNT-Hc 10	TeNT-Hc 15	Mix Ag 15	TT 15

## Data Availability

The data used to support the findings of this study are available from the corresponding author upon request.
